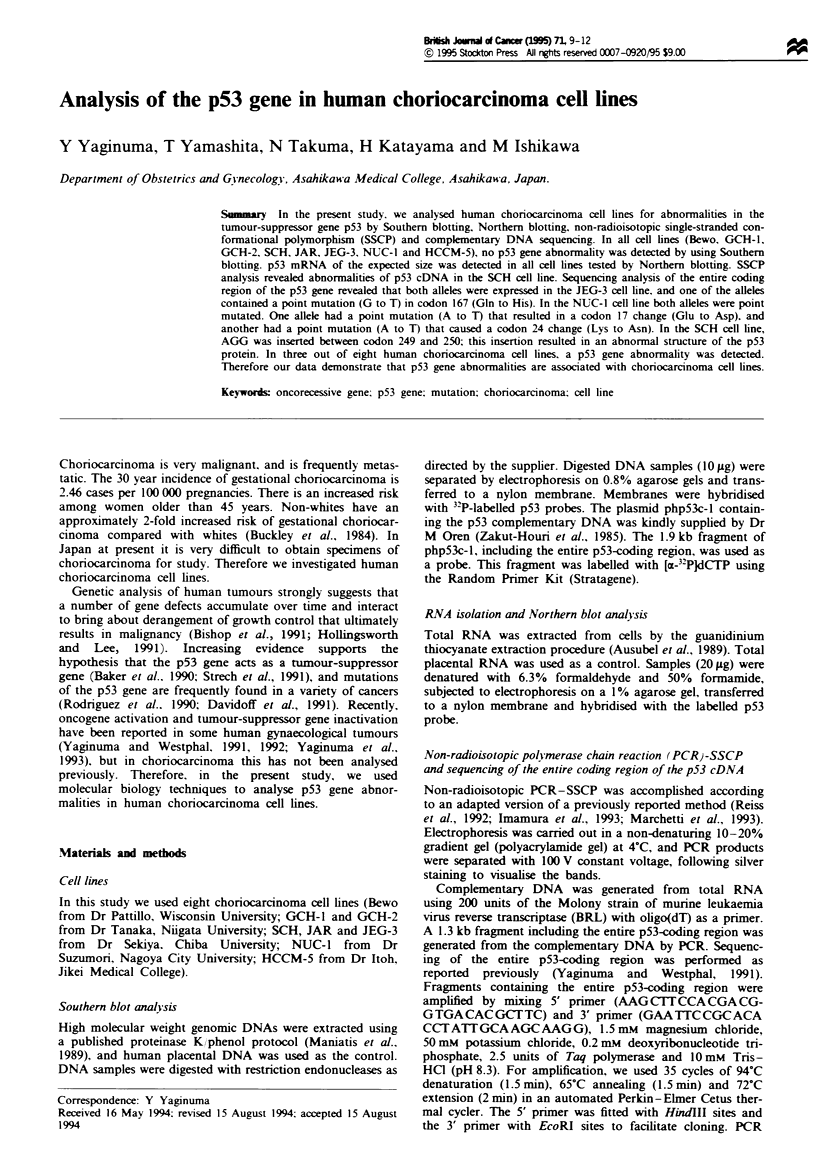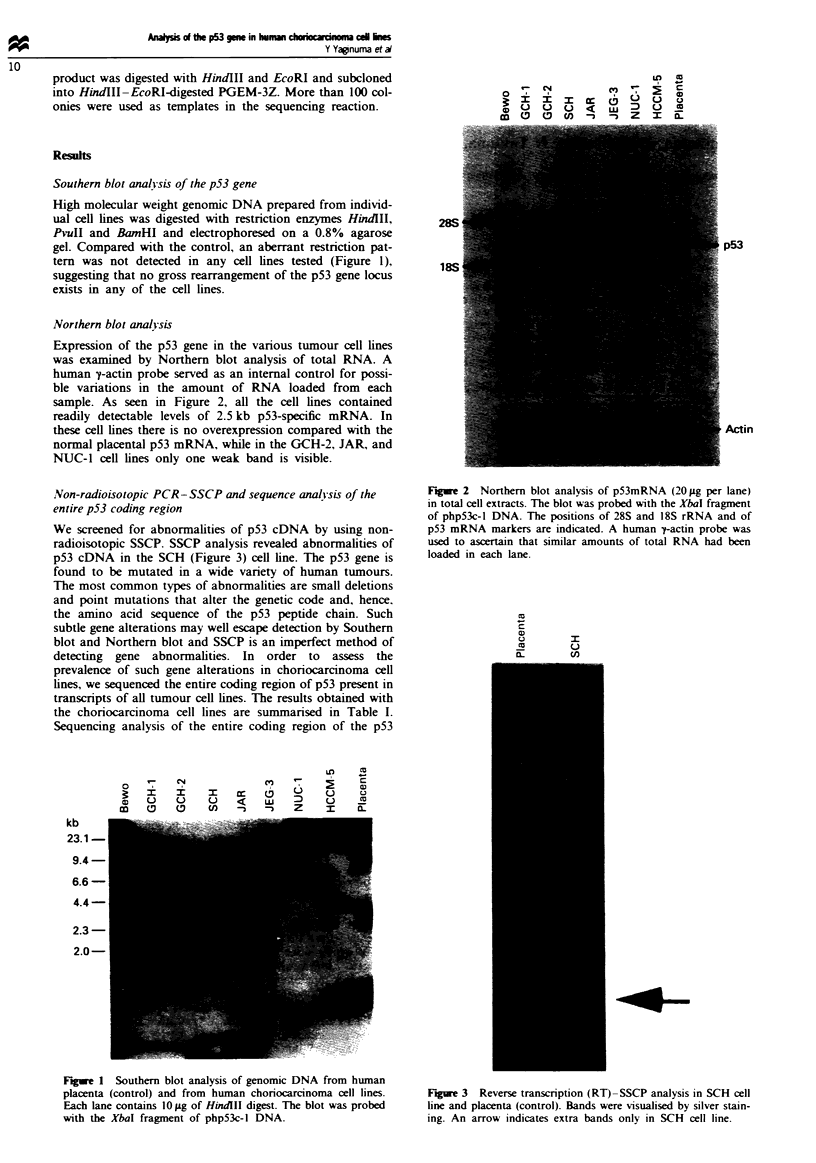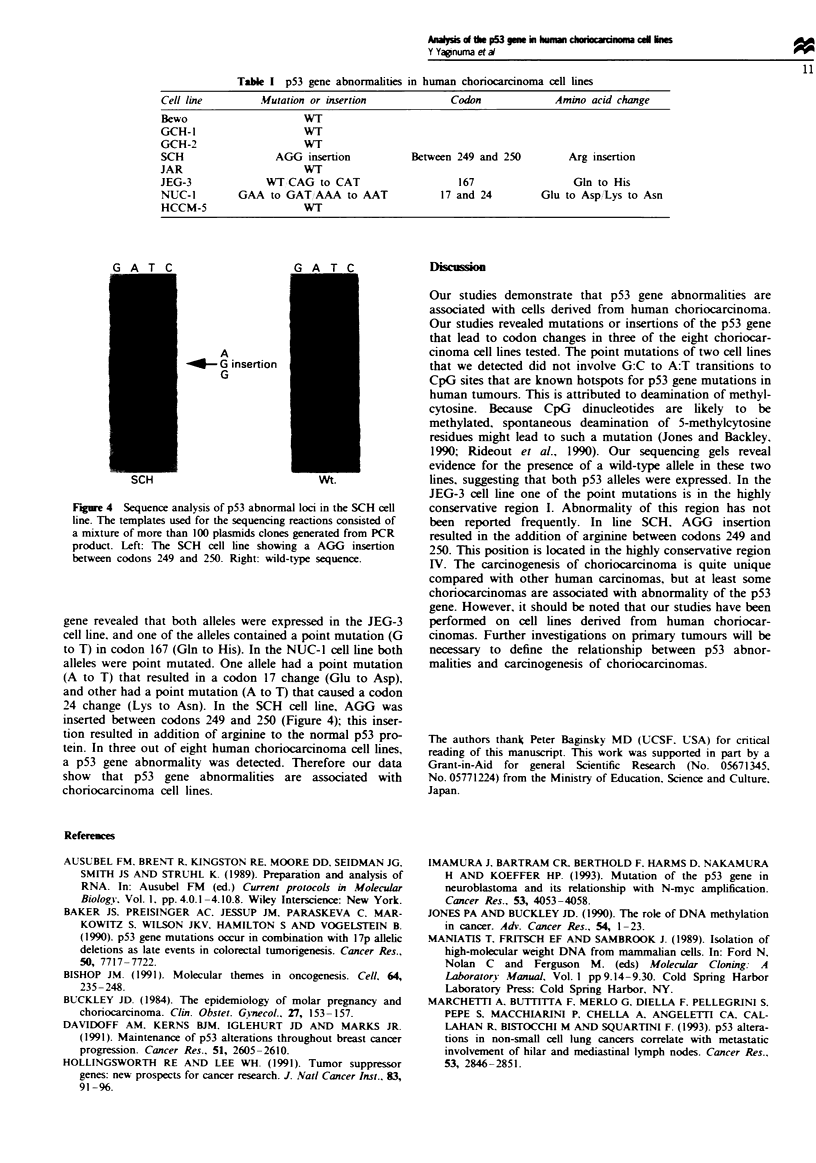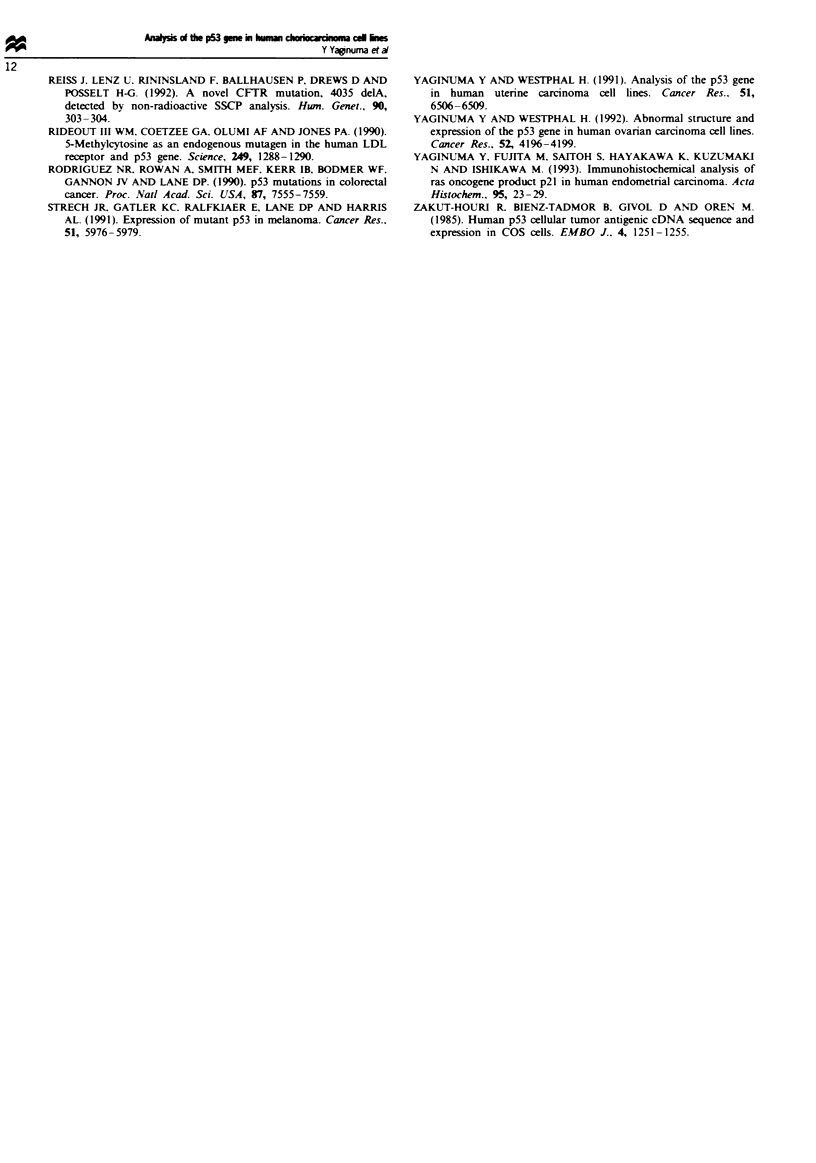# Analysis of the p53 gene in human choriocarcinoma cell lines.

**DOI:** 10.1038/bjc.1995.3

**Published:** 1995-01

**Authors:** Y. Yaginuma, T. Yamashita, N. Takuma, H. Katayama, M. Ishikawa

**Affiliations:** Department of Obstetrics and Gynecology, Asahikawa Medical College, Japan.

## Abstract

**Images:**


British Journal of Cancer (1995) 71, 9-12

© 1995 Stockton Press All rights reserved 0007-0920/95 $9.00

### Analysis of the p53 gene in human choriocarcinoma cell lines

Y Yaginuma, T Yamashita, N Takuma, H Katayama and M Ishikawa

*Department of Obstetrics and Gynecology, Asahikawa Medical College, Asahikawa, Japan.*

**Summary** In the present study, we analysed human choriocarcinoma cell lines for abnormalities in the tumour-suppressor gene p53 by Southern blotting, Northern blotting, non-radioisotopic single-stranded conformational polymorphism (SSCP) and complementary DNA sequencing. In all cell lines (Bewo, GCH-1, GCH-2, SCH, JAR, JEG-3, NUC-1 and HCCM-5), no p53 gene abnormality was detected by using Southern blotting. p53 mRNA of the expected size was detected in all cell lines tested by Northern blotting. SSCP analysis revealed abnormalities of p53 cDNA in the SCH cell line. Sequencing analysis of the entire coding region of the p53 gene revealed that both alleles were expressed in the JEG-3 cell line, and one of the alleles contained a point mutation (G to T) in codon 167 (Gln to His). In the NUC-1 cell line both alleles were point mutated. One allele had a point mutation (A to T) that resulted in a codon 17 change (Glu to Asp), and another had a point mutation (A to T) that caused a codon 24 change (Lys to Asn). In the SCH cell line, AGG was inserted between codon 249 and 250; this insertion resulted in an abnormal structure of the p53 protein. In three out of eight human choriocarcinoma cell lines, a p53 gene abnormality was detected. Therefore our data demonstrate that p53 gene abnormalities are associated with choriocarcinoma cell lines.

**Keywords:** oncorecessive gene; p53 gene; mutation; choriocarcinoma; cell line

Choriocarcinoma is very malignant, and is frequently metastatic. The 30 year incidence of gestational choriocarcinoma is 2.46 cases per 100 000 pregnancies. There is an increased risk among women older than 45 years. Non-whites have an approximately 2-fold increased risk of gestational choriocarcinoma compared with whites (Buckley *et al.*, 1984). In Japan at present it is very difficult to obtain specimens of choriocarcinoma for study. Therefore we investigated human choriocarcinoma cell lines.

Genetic analysis of human tumours strongly suggests that a number of gene defects accumulate over time and interact to bring about derangement of growth control that ultimately results in malignancy (Bishop *et al.*, 1991; Hollingsworth and Lee, 1991). Increasing evidence supports the hypothesis that the p53 gene acts as a tumour-suppressor gene (Baker *et al.*, 1990; Strech *et al.*, 1991), and mutations of the p53 gene are frequently found in a variety of cancers (Rodriguez *et al.*, 1990; Davidoff *et al.*, 1991). Recently, oncogene activation and tumour-suppressor gene inactivation have been reported in some human gynaecological tumours (Yaginuma and Westphal, 1991, 1992; Yaginuma *et al.*, 1993), but in choriocarcinoma this has not been analysed previously. Therefore, in the present study, we used molecular biology techniques to analyse p53 gene abnormalities in human choriocarcinoma cell lines.

### Materials and methods

### *Cell lines*

In this study we used eight choriocarcinoma cell lines (Bewo from Dr Pattillo, Wisconsin University; GCH-1 and GCH-2 from Dr Tanaka, Niigata University; SCH, JAR and JEG-3 from Dr Sekiya, Chiba University; NUC-1 from Dr Suzumori, Nagoya City University; HCCM-5 from Dr Itoh, Jikei Medical College).

### *Southern blot analysis*

High molecular weight genomic DNAs were extracted using a published proteinase K/phenol protocol (Maniatis *et al.*, 1989), and human placental DNA was used as the control. DNA samples were digested with restriction endonucleases as directed by the supplier. Digested DNA samples (10 μg) were separated by electrophoresis on 0.8% agarose gels and transferred to a nylon membrane. Membranes were hybridised with $^{32}$P-labelled p53 probes. The plasmid php53c-1 containing the p53 complementary DNA was kindly supplied by Dr M Oren (Zakut-Houri *et al.*, 1985). The 1.9 kb fragment of php53c-1, including the entire p53-coding region, was used as a probe. This fragment was labelled with [α-$^{32}$P]dCTP using the Random Primer Kit (Stratagene).

### *RNA isolation and Northern blot analysis*

Total RNA was extracted from cells by the guanidinium thiocyanate extraction procedure (Ausubel *et al.*, 1989). Total placental RNA was used as a control. Samples (20 μg) were denatured with 6.3% formaldehyde and 50% formamide, subjected to electrophoresis on a 1% agarose gel, transferred to a nylon membrane and hybridised with the labelled p53 probe.

### *Non-radioisotopic polymerase chain reaction (PCR)-SSCP and sequencing of the entire coding region of the p53 cDNA*

Non-radioisotopic PCR-SSCP was accomplished according to an adapted version of a previously reported method (Reiss *et al.*, 1992; Imamura *et al.*, 1993; Marchetti *et al.*, 1993). Electrophoresis was carried out in a non-denaturing 10-20% gradient gel (polyacrylamide gel) at 4°C, and PCR products were separated with 100 V constant voltage, following silver staining to visualise the bands.

Complementary DNA was generated from total RNA using 200 units of the Molony strain of murine leukaemia virus reverse transcriptase (BRL) with oligo(dT) as a primer. A 1.3 kb fragment including the entire p53-coding region was generated from the complementary DNA by PCR. Sequencing of the entire p53-coding region was performed as reported previously (Yaginuma and Westphal, 1991). Fragments containing the entire p53-coding region were amplified by mixing 5′ primer (AAGCTTCCACGACGGTGACACGCTTC) and 3′ primer (GAATTCCGCACACCTATTGCAAGCAAGG), 1.5 mM magnesium chloride, 50 mM potassium chloride, 0.2 mM deoxyribonucleotide triphosphate, 2.5 units of *Taq* polymerase and 10 mM Tris-HCl (pH 8.3). For amplification, we used 35 cycles of 94°C denaturation (1.5 min), 65°C annealing (1.5 min) and 72°C extension (2 min) in an automated Perkin-Elmer Cetus thermal cycler. The 5′ primer was fitted with *Hin*dIII sites and the 3′ primer with *Eco*RI sites to facilitate cloning. PCR

Correspondence: Y Yaginuma
Received 16 May 1994; revised 15 August 1994; accepted 15 August 1994

product was digested with *Hind*III and *Eco*RI and subcloned into *Hind*III–*Eco*RI-digested PGEM-3Z. More than 100 colonies were used as templates in the sequencing reaction.

### Results

### *Southern blot analysis of the p53 gene*

High molecular weight genomic DNA prepared from individual cell lines was digested with restriction enzymes *Hind*III, *Pvu*II and *Bam*HI and electrophoresed on a 0.8% agarose gel. Compared with the control, an aberrant restriction pattern was not detected in any cell lines tested (Figure 1), suggesting that no gross rearrangement of the p53 gene locus exists in any of the cell lines.

### *Northern blot analysis*

Expression of the p53 gene in the various tumour cell lines was examined by Northern blot analysis of total RNA. A human γ-actin probe served as an internal control for possible variations in the amount of RNA loaded from each sample. As seen in Figure 2, all the cell lines contained readily detectable levels of 2.5 kb p53-specific mRNA. In these cell lines there is no overexpression compared with the normal placental p53 mRNA, while in the GCH-2, JAR, and NUC-1 cell lines only one weak band is visible.

### *Non-radioisotopic PCR–SSCP and sequence analysis of the entire p53 coding region*

We screened for abnormalities of p53 cDNA by using non-radioisotopic SSCP. SSCP analysis revealed abnormalities of p53 cDNA in the SCH (Figure 3) cell line. The p53 gene is found to be mutated in a wide variety of human tumours. The most common types of abnormalities are small deletions and point mutations that alter the genetic code and, hence, the amino acid sequence of the p53 peptide chain. Such subtle gene alterations may well escape detection by Southern blot and Northern blot and SSCP is an imperfect method of detecting gene abnormalities. In order to assess the prevalence of such gene alterations in choriocarcinoma cell lines, we sequenced the entire coding region of p53 present in transcripts of all tumour cell lines. The results obtained with the choriocarcinoma cell lines are summarised in Table I. Sequencing analysis of the entire coding region of the p53

**Figure 1** Southern blot analysis of genomic DNA from human placenta (control) and from human choriocarcinoma cell lines. Each lane contains 10 μg of *Hind*III digest. The blot was probed with the *Xba*I fragment of php53c-1 DNA.

**Figure 2** Northern blot analysis of p53mRNA (20 μg per lane) in total cell extracts. The blot was probed with the *Xba*I fragment of php53c-1 DNA. The positions of 28S and 18S rRNA and of p53 mRNA markers are indicated. A human γ-actin probe was used to ascertain that similar amounts of total RNA had been loaded in each lane.

**Figure 3** Reverse transcription (RT)–SSCP analysis in SCH cell line and placenta (control). Bands were visualised by silver staining. An arrow indicates extra bands only in SCH cell line.

**Table I** p53 gene abnormalities in human choriocarcinoma cell lines

| *Cell line* | *Mutation or insertion* | *Codon* | *Amino acid change* |
|---|---|---|---|
| Bewo | WT | | |
| GCH-1 | WT | | |
| GCH-2 | WT | | |
| SCH | AGG insertion | Between 249 and 250 | Arg insertion |
| JAR | WT | | |
| JEG-3 | WT/CAG to CAT | 167 | Gln to His |
| NUC-1 | GAA to GAT/AAA to AAT | 17 and 24 | Glu to Asp/Lys to Asn |
| HCCM-5 | WT | | |

**Figure 4** Sequence analysis of p53 abnormal loci in the SCH cell line. The templates used for the sequencing reactions consisted of a mixture of more than 100 plasmids clones generated from PCR product. Left: The SCH cell line showing a AGG insertion between codons 249 and 250. Right: wild-type sequence.

gene revealed that both alleles were expressed in the JEG-3 cell line, and one of the alleles contained a point mutation (G to T) in codon 167 (Gln to His). In the NUC-1 cell line both alleles were point mutated. One allele had a point mutation (A to T) that resulted in a codon 17 change (Glu to Asp), and other had a point mutation (A to T) that caused a codon 24 change (Lys to Asn). In the SCH cell line, AGG was inserted between codons 249 and 250 (Figure 4); this insertion resulted in addition of arginine to the normal p53 protein. In three out of eight human choriocarcinoma cell lines, a p53 gene abnormality was detected. Therefore our data show that p53 gene abnormalities are associated with choriocarcinoma cell lines.

### Discussion

Our studies demonstrate that p53 gene abnormalities are associated with cells derived from human choriocarcinoma. Our studies revealed mutations or insertions of the p53 gene that lead to codon changes in three of the eight choriocarcinoma cell lines tested. The point mutations of two cell lines that we detected did not involve G:C to A:T transitions to CpG sites that are known hotspots for p53 gene mutations in human tumours. This is attributed to deamination of methylcytosine. Because CpG dinucleotides are likely to be methylated, spontaneous deamination of 5-methylcytosine residues might lead to such a mutation (Jones and Backley, 1990; Rideout *et al.*, 1990). Our sequencing gels reveal evidence for the presence of a wild-type allele in these two lines, suggesting that both p53 alleles were expressed. In the JEG-3 cell line one of the point mutations is in the highly conservative region I. Abnormality of this region has not been reported frequently. In line SCH, AGG insertion resulted in the addition of arginine between codons 249 and 250. This position is located in the highly conservative region IV. The carcinogenesis of choriocarcinoma is quite unique compared with other human carcinomas, but at least some choriocarcinomas are associated with abnormality of the p53 gene. However, it should be noted that our studies have been performed on cell lines derived from human choriocarcinomas. Further investigations on primary tumours will be necessary to define the relationship between p53 abnormalities and carcinogenesis of choriocarcinomas.

The authors thank Peter Baginsky MD (UCSF, USA) for critical reading of this manuscript. This work was supported in part by a Grant-in-Aid for general Scientific Research (No. 05671345, No. 05771224) from the Ministry of Education, Science and Culture, Japan.

### References

AUSUBEL FM, BRENT R, KINGSTON RE, MOORE DD, SEIDMAN JG, SMITH JS AND STRUHL K. (1989). Preparation and analysis of RNA. In: Ausubel FM (ed.) *Current protocols in Molecular Biology*, Vol. 1, pp. 4.0.1–4.10.8. Wiley Interscience: New York.

BAKER JS, PREISINGER AC, JESSUP JM, PARASKEVA C, MARKOWITZ S, WILSON JKV, HAMILTON S AND VOGELSTEIN B. (1990). p53 gene mutations occur in combination with 17p allelic deletions as late events in colorectal tumorigenesis. *Cancer Res.*, **50**, 7717–7722.

BISHOP JM. (1991). Molecular themes in oncogenesis. *Cell*, **64**, 235–248.

BUCKLEY JD. (1984). The epidemiology of molar pregnancy and choriocarcinoma. *Clin. Obstet. Gynecol.*, **27**, 153–157.

DAVIDOFF AM, KERNS BJM, IGLEHURT JD AND MARKS JR. (1991). Maintenance of p53 alterations throughout breast cancer progression. *Cancer Res.*, **51**, 2605–2610.

HOLLINGSWORTH RE AND LEE WH. (1991). Tumor suppressor genes: new prospects for cancer research. *J. Natl Cancer Inst.*, **83**, 91–96.

IMAMURA J, BARTRAM CR, BERTHOLD F, HARMS D, NAKAMURA H AND KOEFFER HP. (1993). Mutation of the p53 gene in neuroblastoma and its relationship with N-myc amplification. *Cancer Res.*, **53**, 4053–4058.

JONES PA AND BUCKLEY JD. (1990). The role of DNA methylation in cancer. *Adv. Cancer Res.*, **54**, 1–23.

MANIATIS T, FRITSCH EF AND SAMBROOK J. (1989). Isolation of high-molecular weight DNA from mammalian cells. In: Ford N, Nolan C and Ferguson M. (eds) *Molecular Cloning: A Laboratory Manual*, Vol. 1 pp 9.14–9.30. Cold Spring Harbor Laboratory Press: Cold Spring Harbor, NY.

MARCHETTI A, BUTTITTA F, MERLO G, DIELLA F, PELLEGRINI S, PEPE S, MACCHIARINI P, CHELLA A, ANGELETTI CA, CALLAHAN R, BISTOCCHI M AND SQUARTINI F. (1993). p53 alterations in non-small cell lung cancers correlate with metastatic involvement of hilar and mediastinal lymph nodes. *Cancer Res.*, **53**, 2846–2851.

REISS J, LENZ U, RININSLAND F, BALLHAUSEN P, DREWS D AND POSSELT H-G. (1992). A novel CFTR mutation, 4035 delA, detected by non-radioactive SSCP analysis. *Hum. Genet.*, **90**, 303–304.

RIDEOUT III WM, COETZEE GA, OLUMI AF AND JONES PA. (1990). 5-Methylcytosine as an endogenous mutagen in the human LDL receptor and p53 gene. *Science*, **249**, 1288–1290.

RODRIGUEZ NR, ROWAN A, SMITH MEF, KERR IB, BODMER WF, GANNON JV AND LANE DP. (1990). p53 mutations in colorectal cancer. *Proc. Natl Acad. Sci. USA*, **87**, 7555–7559.

STRECH JR, GATLER KC, RALFKIAER E, LANE DP AND HARRIS AL. (1991). Expression of mutant p53 in melanoma. *Cancer Res.*, **51**, 5976–5979.

YAGINUMA Y AND WESTPHAL H. (1991). Analysis of the p53 gene in human uterine carcinoma cell lines. *Cancer Res.*, **51**, 6506–6509.

YAGINUMA Y AND WESTPHAL H. (1992). Abnormal structure and expression of the p53 gene in human ovarian carcinoma cell lines. *Cancer Res.*, **52**, 4196–4199.

YAGINUMA Y, FUJITA M, SAITOH S, HAYAKAWA K, KUZUMAKI N AND ISHIKAWA M. (1993). Immunohistochemical analysis of ras oncogene product p21 in human endometrial carcinoma. *Acta Histochem.*, **95**, 23–29.

ZAKUT-HOURI R, BIENZ-TADMOR B, GIVOL D AND OREN M. (1985). Human p53 cellular tumor antigenic cDNA sequence and expression in COS cells. *EMBO J.*, **4**, 1251–1255.